# New-Onset Diabetes in the Setting of Beta-Cell Dysfunction in a Young Patient With COVID-19 Infection

**DOI:** 10.7759/cureus.16497

**Published:** 2021-07-19

**Authors:** Mohsen S Alshamam, Nso Nso, Zarwa Idrees, Saba Iqbal, Victoria Ghernautan, Vikram Sumbly, Mariam Agladze, Saifullah Khan, Issac Sachmechi

**Affiliations:** 1 Internal Medicine, Ichan School of Medicine at Mount Sinai, New York City (NYC) Health + Hospitals, Jamaica, USA; 2 Internal Medicine, Icahn School of Medicine at Mount Sinai, New York City (NYC) Health + Hospitals Queens, Jamaica, USA; 3 General Medicine, Saint James School of Medicine, St. Vincent, VCT

**Keywords:** covid-19 and diabetes, beta-cell dysfunction, sars-cov-2, post infectious diabetes, covid hyperglycemia

## Abstract

Reciprocal relationships between viral illness and chronic diseases have been established. Such relationships augment one another and increase the potential harm. The coronavirus 2019 pandemic proved that the most vulnerable populations are the ones with underlying chronic diseases, especially diabetes mellitus. As new data are evolving, viral illnesses, like COVID-19, have been speculated to potentially induce diabetes mellitus. Here we report a 20-year-old male with no past medical history who presented with polyuria, polydipsia, and dry mouth. He was found to have significant hyperglycemia. He had COVID-19-like symptoms a few weeks prior to admission and was tested positive for COVID-19, but the symptoms had resolved prior to his presentation. He was managed with intravenous fluids (IVFs), electrolytes replacement, and insulin. He was diagnosed with new-onset diabetes mellitus likely secondary to a recent COVID-19 infection and was discharged home on insulin, oral antidiabetic medications, and outpatient follow-up with primary care clinic and endocrinology clinic.

## Introduction

Coronavirus disease 2019 (COVID-19) pandemic has affected the whole world and the data regarding social, economic, and health impacts are rapidly evolving. Many studies have shown that diabetes mellitus (DM) is associated with increased morbidity and mortality in COVID-19 infection [1,2]. Recent studies have raised the concern about the relationship of COVID-19 with new-onset DM in the setting of beta-cell dysfunction. Given the evolving nature of such phenomena, it is difficult to ascertain the exact incidence or prevalence at this time. Also, cases of uncontrolled hyperglycemia and ketoacidosis in the setting of COVID-19 infection are being reported increasingly [3]. Here, we present a case of a young patient with COVID-19 infection who presented with hyperglycemia and was found to have diabetes of unclear type. Beta-cell dysfunction, in this case, is evident given low c-peptide levels.

## Case presentation

A 20-year-old white male with no significant past medical history was referred from an urgent care center for a high blood glucose reading. The patient has been experiencing polydipsia, polyurea, and severe dry mouth to the point that his tongue bleeds after brushing teeth. Approximately four weeks before the presentation, he experienced COVID-19 symptoms including fevers, myalgias, fatigue, and ageusia (inability to taste) was tested positive for COVID-19. All symptoms resolved prior to this hospital visit. He lost approximately 20 pounds over the last five months, which he attributed to intermittent fasting. His review of systems was otherwise negative. His family history was significant for type 2 DM (T2DM) in two of his grandparents but negative for any autoimmune disorders. He has no known allergies. He admits to smoking marijuana and tobacco (two cigarettes per week), and drinking alcohol (drinks four beers daily), but denies any other drug use.

His vital signs were within normal limits and the rest of his physical exam was unremarkable. His body mass index (BMI) was found to be 22.5 kg/m^2^ (reference range 18.5-24.9 kg/m^2^). Lab work was significant for hyperglycemia, hyponatremia, an anion gap of 13, normal pH, glucosuria, and ketonuria but negative for ketonemia (Table 1). Also, he was found to have a high hemoglobin A1c, a low c-peptide, and negative insulin antibodies (Table 1). His differential diagnosis includes new-onset DM secondary to COVID-19 due to beta-cell dysfunction, T2DM, or type 1 DM (T1DM). Transient hyperglycemia secondary to COVID-19 as well as undiagnosed DM prior to COVID-19 infection were other possibilities. The patient responded well to six liters of intravenous (IV) fluids, 15 units of insulin Levemir, seven units of insulin lispro, four units of insulin regular, oral and IV potassium supplementation. He was discharged home with five units of insulin Levemir nightly, Janumet 50-500 mg twice daily, and follow-ups with primary care and endocrinology as an outpatient but he never did.

**Table 1:** Patient’s pertinent labs. pH obtained from a venous blood gas.

Lab Name	Value	Reference Range (RR)
Glucose	539	70-105 mg/dL
Sodium (Na)	132	136-145 mmol/L
Potassium (K)	4.2	3.5-5.1 mmoL/L
Osmolality, Serum	314	275-290 mOsm/kg
Bicarbonate (HCO_3_)	28	22-29 mmol/L
pH	7.44	7.32-7.43
Blood Urea Nitrogen (BUN)	12	6-23 mg/dL
Creatinine (Cr)	1.03	0.7-1.2 mg/dL
Anion Gap	13	8-16 mEq/L
Lactate	1.6	0.6-1.4 mmol/L
Hemoglobin A1c	13.7	4%-5.6%
C-peptide	0.6	1.1-4.4 ng/mL
Insulin Antibodies	<0.4	<0.4 U/mL
Ketones, Blood	Negative	Negative
Urine Glucose	=/>1,000	Negative
Urine Specific Gravity	=/>1.030	1.005-1.030
Ketones, Urine	80	Negative
COVID-19 Test	Negative	Negative

## Discussion

Recent studies have hypothesized that DM is linked to inappropriate beta-cell number/function due to beta-cell dysfunction or apoptosis which are both postulated in the pathogenesis of T2DM [4]. In the form of T2DM in COVID-19 infected patients several mechanisms were entertained including activation of the Renin-Angiotensin-Aldosterone System (RAAS) which leads to the imbalance between angiotensin-2 (AT-2) and angiotensin-converting enzyme-2 (ACE-2). While AT-2 is said to be prooxidant, pro-constrictive, hypertrophic, proinflammatory, proliferative, and profibrotic; ACE-2 is exactly the opposite [4]. Therefore, it is thought that RAAS activation happens via SAR-CoV-2 binding to ACE-2 in pancreatic islet cells, diminishing its protective ability and activating the systemic RAAS (cRAAS) and localized tissue RAAS (tRAAS) existing on the epithelial ductal cells of the exocrine pancreas [4]. This cascade leads to increased levels of AT-2 and decreased numbers of ACE-2 favoring oxidative stress, islet inflammation, fibrosis, amyloid deposition, and apoptosis ending in islet cells damage and death [4]. ACE-2 degrades AT-2 into vasodilatory and anti-inflammatory products in normal circumstances; however, in COVID-19 infection ACE-2 is mainly occupied by SARS-CoV-2 leaving behind AT-2 uncleaved [5].

There have also been postulations regarding the potential of new-onset T1DM triggered by COVID-19 [6]. Similar mechanisms have been proposed including, but not limited to, B-cell destruction, inflammation, and changes in healthcare behavior secondary to the pandemic [7]. SARS-CoV-2 can trigger severe diabetic ketoacidosis (DKA) at presentation in individuals with new-onset T1DM. However, there is no hard evidence that SARS-CoV-2 induces T1DM on its own accord. The COVID-19 pandemic might have altered diabetes presentation and DKA severity [6]. The lockdown implemented in some countries has significantly affected T1DM and led to an increased DKA frequency, including children with new-onset T1DM, potentially owing to delayed presentation. Long-term follow-up of children and adults presenting with new-onset diabetes during this pandemic is required to fully understand the type of diabetes induced by COVID-19 [8].

In our patient, there were no concerning risk factors for DM, and his clinical presentation was within an acceptable time frame of his COVID-19 infection, making it the basis for the argument that COVID-19 triggered new-onset DM in this patient. His workup was consistent with decreased insulin secretion from pancreatic beta-cells indicated by the low c-peptide levels, which points toward beta-cell dysfunction or destruction rather than insulin resistance. While it is not clear yet/early to determine the type of diabetes this patient had developed, some of the clues pointing toward T1DM are lean BMI, young age at presentation, and swift insulin response with low insulin requirement arguing against insulin resistance as a cause of hyperglycemia in this patient. On the contrary, family history of diabetes, as well as the use of oral medications for diabetes at discharge, speaks in favor of T2DM. Therefore, regular follow-ups and further workup, i.e anti-glutamic acid decarboxylase antibodies (GADA) and others (like IA-2A, IA-2BA, Zn-T8A), are needed to execute a just verdict as to which of the two has resulted from COVID-19 in this case.

## Conclusions

Of the many complications of the COVID-19 virus, DM is a rare but possible complication. A thorough history should be obtained to exclude any confounders, and workup should be broadened to identify the type of DM. This is important to gauge the patient’s long-term need for insulin, as the management of the two types of DM is somewhat different. This rare complication of COVID-19 is known to be one of the most costly comorbidities at the national and international levels, which necessitates the need for diligent isolative practices as well as early vaccination when possible.
